# Characteristics of progressive multifocal leukoencephalopathy clarified through internet-assisted laboratory surveillance in Japan

**DOI:** 10.1186/1471-2377-12-121

**Published:** 2012-10-15

**Authors:** Kazuo Nakamichi, Hidehiro Mizusawa, Masahito Yamada, Shuji Kishida, Yoshiharu Miura, Toshio Shimokawa, Tomohiko Takasaki, Chang-Kweng Lim, Ichiro Kurane, Masayuki Saijo

**Affiliations:** 1 Department of Virology 1, National Institute of Infectious Diseases, Toyama, Shinjuku-ku, Tokyo, 162-8640, Japan; 2 Department of Neurology and Neurological Science, Graduate School, Tokyo Medical and Dental University, Bunkyo-ku, Tokyo, 113-8519, Japan; 3 Department of Neurology and Neurobiology of Aging, Kanazawa University Graduate School of Medical Science, Ishikawa, Kanazawa, 920-8640, Japan; 4 Division of Neurology, Tokyo Metropolitan Cancer and Infectious diseases Center Komagome Hospital, Bunkyo-ku, Tokyo, 113-8677, Japan; 5 Department of Ecosocial System Engineering, Interdisciplinary Graduate School of Medicine and Engineering, University of Yamanashi, Kofu, Yamanashi, 400-8511, Japan

**Keywords:** Cerebrospinal fluid, Surveillance, Japan, JC virus, Progressive multifocal leukoencephalopathy

## Abstract

**Background:**

Progressive multifocal leukoencephalopathy (PML), a rare but fatal demyelinating disease caused by JC virus (JCV), occurs mainly in immunocompromised patients. As PML develops in individuals with various underlying disorders sporadically and infrequently, a nationwide survey of PML is difficult. This study was conducted to elucidate the characteristics of PML in Japan through an internet-assisted laboratory surveillance program.

**Methods:**

A diagnostic support system for PML was established using a real-time PCR assay of JCV DNA in cerebrospinal fluid (CSF), and requests for testing were received from clinicians via specialized websites. Medical histories of patients were collected through standardized questionnaires, and a database of CSF JCV loads and clinical information was created and analyzed.

**Results:**

For 4 years from April 2007 to March 2011, CSF specimens from 419 patients were tested. Forty-eight individuals were found positive for JCV DNA in their CSF and were diagnosed with PML. PML primarily occurred not only in HIV-positive patients (33.3%) but also in patients with hematologic disorders after receiving stem cell transplantation, chemotherapy, and/or immunosuppressive treatment (39.6%). The frequencies of PML cases among the subjects in these two categories were 20.3% and 23.5%, respectively. Although no significant features were observed with respect to CSF JCV loads in PML patients with an HIV infection or hematologic disorder, males were predominant in both groups (100% and 89.5%, respectively). The proportion of PML cases with autoimmune disorders (6.3%) or solid-organ transplants (2.1%) was smaller than those with HIV infection or hematologic disorders, probably due to the limited availability of therapeutic monoclonal antibodies and transplantation from brain dead donors.

**Conclusions:**

The results suggest that the internet-assisted laboratory surveillance program might be a useful strategy for collecting precise real-time information on PML on a national level. The current database provides important background information for the diagnosis and treatment of patients with risk factors for PML.

## Background

Progressive multifocal leukoencephalopathy (PML) is a rare but fatal demyelinating disease caused by JC virus (JCV), a small DNA virus belonging to the family *Polyomaviridae*, genus *Polyomavirus*[1-3]. JCV establishes a persistent and asymptomatic infection in a large number of individuals, with the serologically positive rate for JCV among the adult human population being 50–90%. However, in some severely immunocompromised patients, JCV reactivates and causes a lytic infection in the oligodendrocytes, leading to PML [1-4]. PML develops in human immunodeficiency virus (HIV)-positive patients as well as in those with immunodeficiency due to hematological malignancies, chemotherapy, transplantation, lymphocyte depletion or autoimmune disorders, such as systemic lupus erythematosus (SLE), treated with immunosuppressive agents [1-4]. In addition, PML has recently been diagnosed in patients receiving immunomodulatory therapies with monoclonal antibodies, such as natalizumab, rituximab, and efalizumab [2,5].

To conduct a nationwide survey of PML, the collection and analysis of large amounts of clinical data are necessary. However, as PML develops in patients with various underlying disorders sporadically and infrequently, a comprehensive surveillance of PML is difficult. Recent epidemiological data on PML were mainly obtained from retrospective cohort studies of HIV-infected persons [6-15] or reported cases of serious adverse events following treatment with monoclonal antibodies [16-23]. Although the results of these investigations are important for a better understanding of PML in association with HIV infection or monoclonal antibody therapies, it is difficult to comprehensively monitor PML patients with diverse underlying diseases. Several other recent studies demonstrated the incidence of PML using national databases in the USA, such as the National Multiple Cause of Death Data system, the Nationwide Inpatient Sample, the US health insurance claims database, and the US Renal Data System [24-27]. While these database screening strategies are considered to be beneficial for the surveillance of PML, the amount of information available for each case is limited.

The detection of JCV DNA in cerebrospinal fluid (CSF) by PCR is a reliable and less-invasive marker for the diagnosis of PML [1]. In Japan, the ongoing CSF testing for JCV DNA has been supported by the Laboratory of Neurovirology, Department of Virology 1, National Institute of Infectious Diseases (NIID), Tokyo, Japan, since April 2007. Through this practice, datasets of clinical information are being obtained not only from PML patients but also from individuals suspected of having PML via their physicians. The current study has been undertaken to assess the occurrence and characteristics of PML patients in Japan over the past 4 years.

## Methods

### Clinical information on patients from whom CSF specimens were collected

This study was performed under informed consent from patients or their family members and with the approval of the Ethical Committee for Biomedical Science in the NIID. CSF testing for JCV DNA was requested from the patients’ physicians for the diagnosis or management of PML primarily via two specialized web sites (available in Japanese language only), [ http://www0.nih.go.jp/vir1/NVL/Virus1/NVL3%20HP/index11.html] and [ http://prion.umin.jp/pml/virus.html]. This diagnostic support system was funded by the Japanese government (Research Committee of Prion Disease and Slow Virus Infection, the Ministry of Health, Labour and Welfare, Japan) and performed free of charge to patients. CSF specimens were collected by lumbar puncture from patients suspected of having PML on the basis of neurological symptoms and/or magnetic resonance imaging (MRI) patterns, and were transferred to the NIID. Patient information including age, sex, underlying disease, and past medical history was collected anonymously through standardized questionnaires.

### Real-time PCR testing for JCV DNA

Total DNAs were extracted from CSF specimens using a QIAamp DNA Blood Mini Kit (Qiagen, Valencia, CA) and subjected to real-time PCR assay as described previously [28]. The pBR322-based plasmid pJC1-4->pJCV containing the complete genome sequence of JCV Mad-1 strain [29] was supplied by the Health Science Research Resources Bank (Osaka, Japan) and was used as the standard DNA for real-time PCR. For clinical testing of JCV DNA in CSF specimens, three different real-time PCR assays were developed to detect either the JCV T or viral protein 1 (VP1) genes and to monitor the contamination of each clinical sample with standard DNA. The primers and TaqMan probe targeting the JCV large T gene were described in an earlier report [28]. A pair of primers (5’- AAT GCA ACA GTG CAA TCT CA -3’ and 5’- GGC CCA ACA CCA AAT TCA TC -3’) and a TaqMan probe (5’- TTG GGT TCC TGA TCC CAC CAG -3’) were designed to detect a highly conserved region within the JCV VP1 gene. The sequences of these primers and probes were 100% identical to the corresponding region of over 360 JCV isolates of various origins in GenBank (data not shown). To control the contamination of samples with standard DNA, another set of primers (5’- CAC AGC TTG ACT GAG GAA -3’ and 5’- GAT GTC GGC GAT ATA GGC -3’) and probe (5’- ATC CTC TAC GCC GGA CGC AT -3’) were also designed to detect the boundary sequence of the JCV genome and pBR322 within pJC1-4->pJCV [see Additional file 1. This primer/probe set detects standard DNA but not the JCV genome. All TaqMan probes were 5’ labeled with 6-carboxyfluorescein and 3’ labeled with Black Hole Quencher-1. These three PCRs were carried out independently for each sample under the conditions described previously [28] except that the annealing temperature was 58°C instead of 60°C. The copy numbers of the viral genome in JCV DNA-positive samples were determined as reported earlier [28]. In each PCR, target DNAs were detected in a range from 1 x 10^9^ to 4 copies per reaction [see Additional file 2 and no amplification signals were detected, even in the presence of high concentrations (over 10^7^ copies) of other polyomaviruses, including BK virus and simian virus 40 (data not shown).

### Statistical analysis

The proportions of JCV-positive or combination antiretroviral therapy (cART)-treated patients in each group were statistically compared by means of a two-tailed Fisher's exact test. For multiple testing, the resulting *P*-value was corrected using the Benjamini-Hochberg method [30]. The numbers of clusters of differentiation 4 (CD4)-positive T cells in JCV-positive and -negative patient groups were compared using a Mann-Whitney U test. The amounts of CSF JCV DNA in different patient groups were compared using a Steel-Dwass nonparametric multiple comparison method. All *P*-values less than 0.05 were judged to be statistically significant.

## Results

### Detection of JCV DNA in CSF specimens from patients

**Figure 1:**
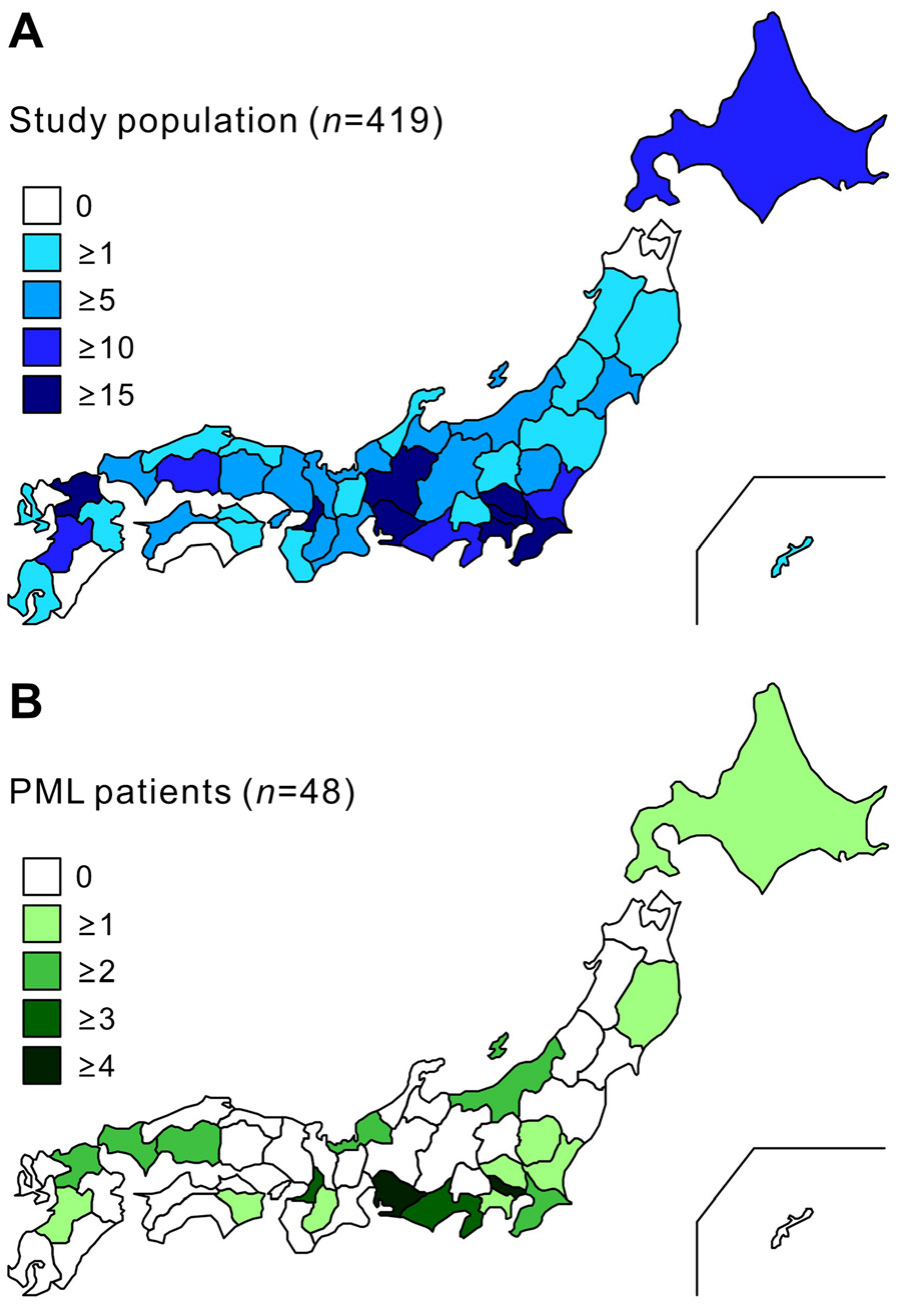
**The geographical distribution patterns of the examined population (A) and PML patients (B).** All prefectures are colored based on the number of individuals.

From April 2007 to the end of March 2011, 504 CSF specimens from 419 patients were submitted to the NIID for testing by hospitals in 43 of Japan’s 47 prefectures (91.5%), with many requests received from the Tokyo metropolitan area and other regions with large populations (Figure 1A). Forty-eight individuals showed a positive reaction for JCV DNA in the real-time PCR targeting the T and VP1 genes and were subsequently diagnosed with PML (Figure 1B). The distribution pattern of PML patients resembled that of the total population. The PCR testing results and underlying diseases of the subjects are summarized in Table 1. Of 48 patients positive for CSF JCV DNA, 16 (33.3%) had HIV infection. The total number of HIV-positive subjects was 79 (20.3%). The proportion of cART-received patients found to be positive and negative for CSF JCV DNA at the initial testing was 31.3% (*n* = 14) and 50.8% (*n* = 57), respectively. The median peripheral blood CD4 counts in the two groups were 37.0 cells / μL (*n* = 13; range, 8–232 cells / μL) and 43.5 cells / μL (*n* = 60; range, 1–400 cells / μL), respectively. For both parameters, no statistical differences were observed between the JCV-positive and -negative patient groups. Nineteen of the JCV-positive patients (39.6%) had hematologic disorders, and the frequency of PML cases in this category was approximately 24%. Of 50 subjects with autoimmune disorders, 3 with SLE were positive for CSF JCV. Among patients with other underlying diseases, 9 JCV-positive cases were observed, and high positive ratios were found among those with lung disease (66.7%) and sarcoidosis (100%). Among these 4 categories of underlying disorders, the proportion of JCV-positive patients with hematologic disorders, but not that of patients with HIV infection, was significantly higher than that of patients with autoimmune disorders (*P* = 0.009). The underlying illness of the remaining 1 JCV-positive patient could not be determined. These results indicate that PML occurs primarily in patients not only with HIV infection but also with hematologic disorders.

**Table 1:** Underlying diseases of patients positive and negative for JCV DNA on the basis of CSF specimens

**Underlying disease**		**No. (%) of JCV-positive patients**		**No. (%) of JCV-negative patients**	
**Category**	**Subcategory**				
HIV infection / AIDS		16	(20.3)	63	(79.7)
Hematologic disorders		19	(23.5)	62	(76.5)
	Acute myeloid leukemia	2	(16.7)	10	(83.3)
	Acute lymphocytic leukemia	1	(14.3)	6	(85.7)
	Chronic myeloid leukemia	0	(0)	2	(100)
	Chronic lymphocytic leukemia	1	(50.0)	1	(50.0)
	Adult T-cell leukemia	1	(50.0)	1	(50.0)
	Hodgkin's lymphoma	1	(50.0)	1	(50.0)
	Non-Hodgkin's lymphoma	6	(22.2)	21	(77.8)
	Aplastic anemia	2	(50.0)	2	(50.0)
	Primary immunodeficiency syndrome	2	(25.0)	6	(75.0)
	Multiple myeloma	2	(66.7)	1	(33.3)
	Primary macroglobulinemia	1	(100)	0	(0)
	Myelodysplastic syndrome	0	(0)	6	(100)
	Other	0	(0)	5	(100)
Autoimmune disorders		3	(6.0)	47	(94.0)
	Multiple sclerosis	0	(0)	11	(100)
	Rheumatoid arthritis	0	(0)	7	(100)
	Systemic lupus erythematosus	3	(20.0)	12	(80.0)
	Other	0	(0)	17	(100)
Other diseases		9	(10.0)	81	(90.0)
	Cerebrovascular disorders	0	(0)	7	(100)
	Solid-organ cancer	0	(0)	13 ^a^	(100)
	Lung diseases	2	(66.7)	1	(33.3)
	HCV-related liver disease	3	(37.5)	5	(62.5)
	Renal diseases	0	(0)	11 ^b^	(100)
	Sarcoidosis	3	(100)	0	(0)
	Other	1 ^c^	(2.2)	44 ^d^	(97.8)
None/Unknown		1	(0.8)	118	(99.2)
Total		48	(11.5)	371	(88.5)

*HCV*, hepatitis C virus.

^a^ Two patients underwent liver transplantation.

^b^ Six patients underwent renal transplantation.

^c^ The patient receiving the liver transplant had common variable immunodeficiency.

^d^ One patient received heart and kidney transplants.

### Characteristics of PML patients with hematologic disorders or other underlying diseases

**Figure 2:**
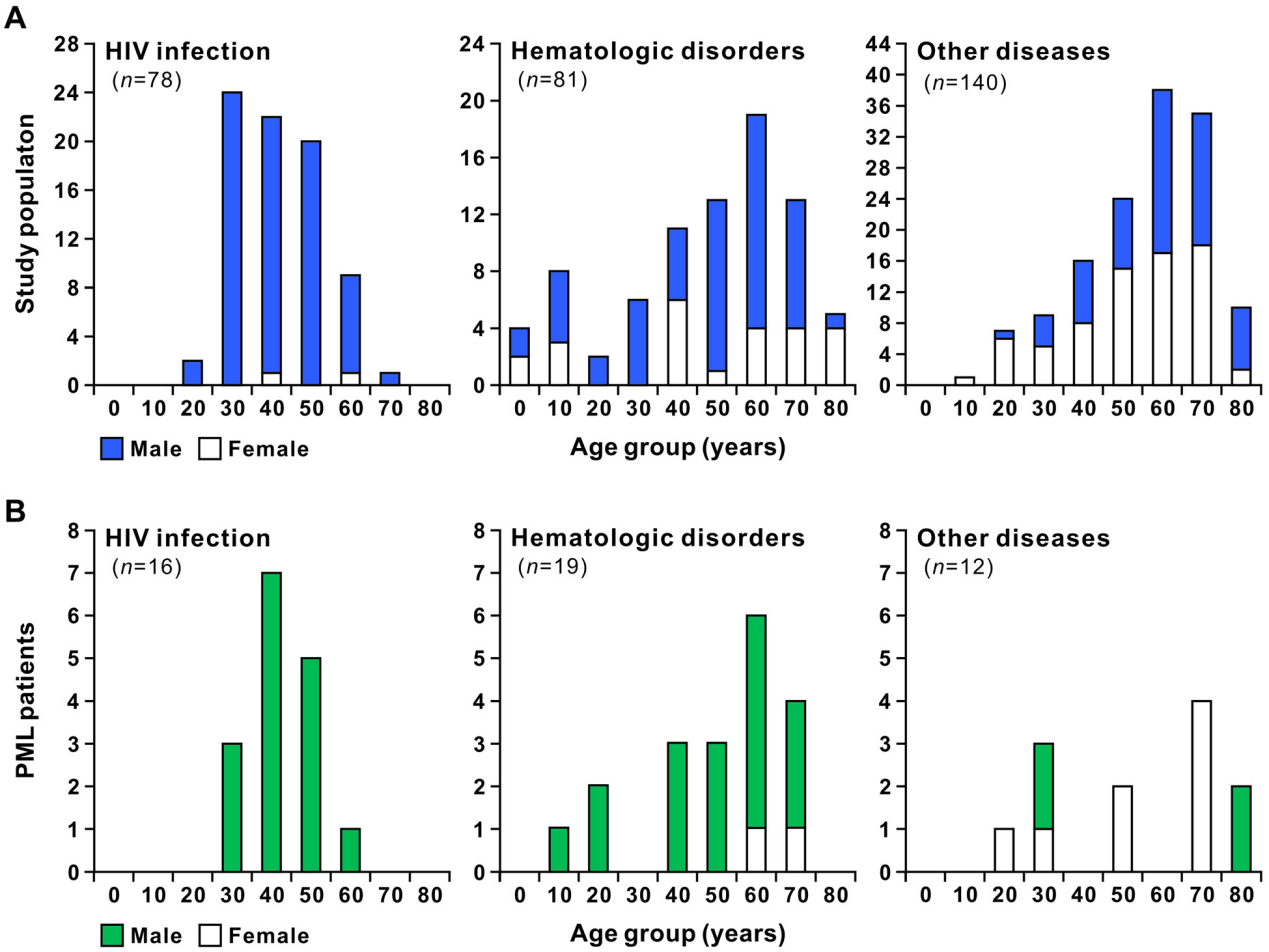
**The age and sex distribution patterns of the study population (A) and PML patients (B).** The subjects and PML patients were divided into three groups on the basis of HIV infection, hematologic disorders, or other underlying diseases including autoimmune disorders. The vertical axes indicate the number of individuals. The solid and open bars show the results for males and females, respectively. The numbers of below the bars represent 10-year age groups (e.g., “0” indicates individuals aged 0 to 9). The data exclude 1 HIV-positive subject whose age was not known.

**Figure 3:**
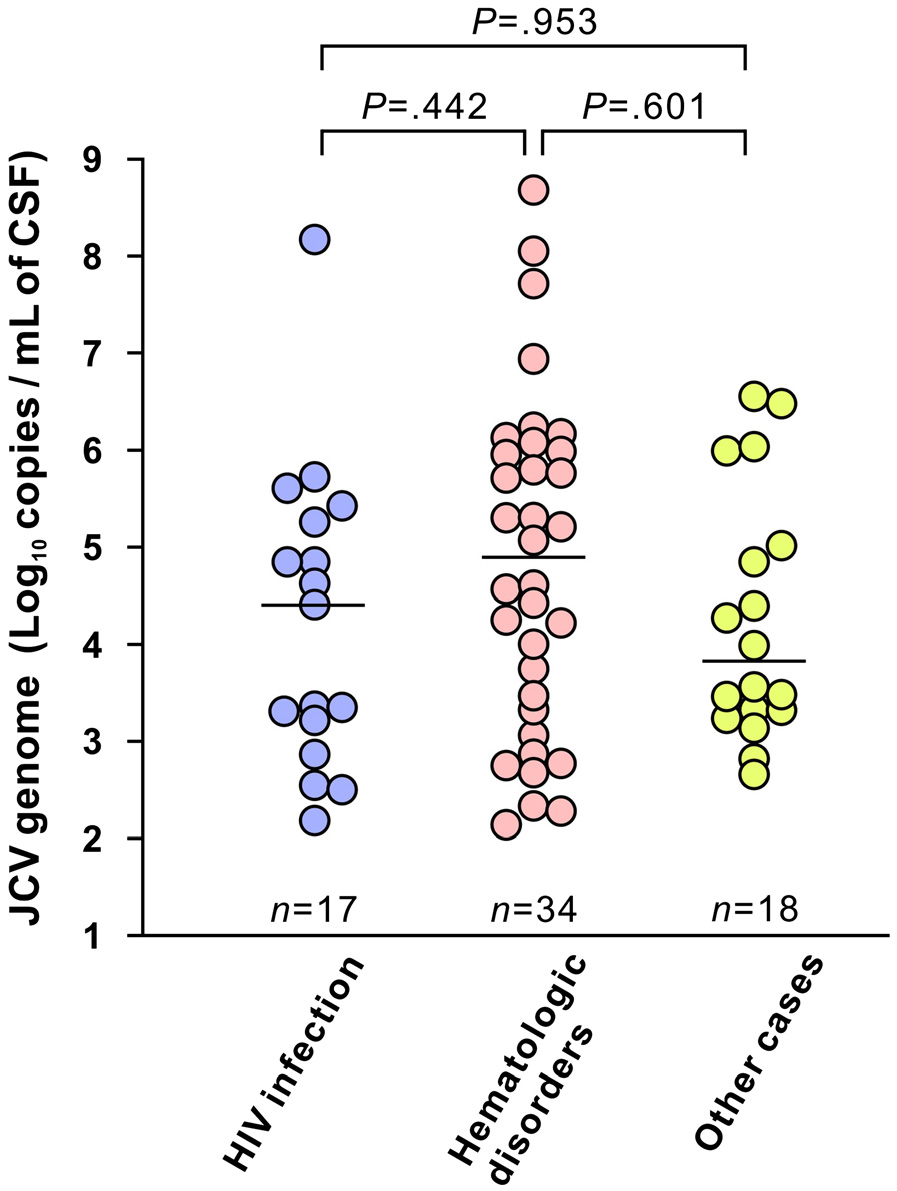
**The JCV DNA levels in the CSF specimens from PML patients.** The CSF viral loads for patients with HIV infection, hematologic disorders, or other diseases are shown (left to right). The open circles indicate the copy number of JCV DNA in each sample, and the horizontal lines represent the medians. In several cases, JCV DNA measurement was performed for different specimens from the same patient during the follow-up period.

Having shown that PML is frequently seen in patients with hematologic disorders in Japan, the characteristics of these patients were compared to those of patients with HIV infection or other non-HIV-related diseases. The age and sex distributions of study population and PML patients are shown in Figure 2. The majority of subjects and PML patients with HIV infection were male in their thirties to sixties, and subjects and PML patients with hematologic disorders or other underlying diseases were found at various ages. PML patients with hematologic disorders were mainly males, while non-HIV-related PML occurred in both sexes (Figure 2B). These data suggest that there is a predominance of males among PML patients; not only among those with HIV infection but also those with hematologic disorders. The median viral loads in CSF specimens from patients with HIV infection, hematologic disorders, and other diseases were 2.6 x 10^4^, 8.0 x 10^4^, and 6.4 x 10^3^ copies per mL, respectively (Figure 3). No statistical differences in the CSF JCV levels were found between the groups. These results indicate that PML cases with hematologic disorders exhibit no significant differences with respect to CSF JCV loads.

### Medical history of PML patients with hematologic disorders

The final set of analyses was conducted to assess the clinical background of PML patients with hematologic disorders. The practice of hematopoietic stem cell transplantation (HSCT) in patients found to be positive or negative for JCV DNA on the basis of CSF specimens is shown in Table 2. Eight of 19 JCV-positive patients with hematological disorders (42.1%) received HSCT for the treatment of acute myeloid leukemia (AML), acute lymphoblastic leukemia (ALL), Hodgkin’s lymphoma (HL), non-Hodgkin’s lymphoma (NHL), aplastic anemia (AA), or multiple myeloma (MM). Among the CSF JCV-negative cases, 19 of 49 individuals (38.8%) received HSCT. There was no statistically significant difference in the proportion of HSCT-treated individuals between the JCV-positive and -negative patients. The characteristics of 8 PML patients receiving HSCT are summarized in Table 3. In these 8 cases, JCV DNA was detected in the CSF specimens at a median of 471.5 days after transplantation (range, 279–833 days). These results indicate that in cases of PML with hematologic disorders, a high proportion of patients had a history of HSCT, and that PML occurred 1–2 years after the various types of transplantation. However, 11 of 19 PML patients with hematologic disorders (57.9%) had no record of HSCT. Thus, potential risk factors for PML were examined in these patients. Table 4 shows the characteristics of PML patients without HSCT. Nine of 11 patients (81.8%) were over 60 years old, and 8 of 11 patients had received combination chemotherapy, cyclophosphamide or rituximab. The remaining 3 individuals had no history of treatment with chemotherapeutic or immunosuppressive agents, but 2 had primary immunodeficiency syndrome without need for these treatments. These data indicate that PML occurred in patients with hematologic disorders not only after HSCT but also after treatment with chemotherapeutic or immunosuppressive drugs.

**Table 2:** Hematopoietic stem cell transplantation (HSCT) in patients positive or negative for CSF JCV

**Underlying disease**	**Proportion (%) of JCV-positive patientswith HSCT (*n*=27)**		**Proportion (%) of JCV-negative patientswith HSCT (*n*=68)**	
Acute myeloid leukemia	2/2	(100)	9/10	(90.0)
Acute lymphoblastic leukemia	1/1	(100)	4/6	(66.7)
Chronic lymphocytic leukemia	0/1	(0)	0/1	(0)
Adult T-cell leukemia	0/1	(0)	0/1	(0)
Hodgkin's lymphoma	1/1	(100)	0/1	(0)
Non-Hodgkin's lymphoma	1/6	(16.7)	5/21	(23.8)
Aplastic anemia	2/2	(100)	0/2	(0)
Primary immunodeficiency syndrome	0/2	(0)	1/6	(16.7)
Multiple myeloma	1/2	(50.0)	0/1	(0)
Primary macroglobulinemia	0/1	(0)	0/0	NA
Total	8/19	(42.1)	19/49	(38.8)

NA, not applicable.

**Table 3:** Characteristics of PML patients with a history of hematopoietic stem cell transplantation

**Patient**	**Age**	**Sex**	**Underlying disease**	**Type of transplant**	**Interval (Days)**^**a**^
1	50	M	Acute myeloid leukemia	Allogeneic bone marrow	279
2	44	M	Acute lymphoblastic leukemia	Allogeneic bone marrow	442
3	42	M	Aplastic anemia	Allogeneic bone marrow	614
4	58	M	Aplastic anemia	Allogeneic bone marrow	493
5	43	M	Acute myeloid leukemia	Umbilical cord blood	450
6	16	M	Hodgkin's lymphoma	Autologous bone marrow	285
7	61	M	Non-Hodgkin's lymphoma	Autologous peripheral blood	775
8	52	M	Multiple myeloma	Autologous peripheral blood	833

*M*, male.

^a^ Days between transplantation and the initial testing for JCV DNA in CSF specimens.

**Table 4:** Chemotherapy or immunosuppressive treatment in PML patients without hematopoietic stem cell transplantation

**Patient**	**Age**	**Sex**	**Underlying disease**	**Chemotherapeutic or immunosuppressive agents**
9	78	M	Non-Hodgkin's lymphoma	CPA, THP, VDS, PSL, R
10	66	M	Non-Hodgkin's lymphoma	CPA, DXR, VCR, PSL, R
11	72	F	Non-Hodgkin's lymphoma	CPA, DXR, VCR, PSL, THP, R
12	64	F	Non-Hodgkin's lymphoma	CPA, DXR, VCR, PSL, ETP
13	77	M	Adult T-cell leukemia	CPA, DXR, VCR, PSL
14	64	M	Multiple myeloma	DEX, DXR, VCR, Bzb
15	67	M	Chronic lymphocytic leukemia	CPA
16	71	M	Primary macroglobulinemia	R
17	62	M	Non-Hodgkin's lymphoma	NA
18	22	M	Primary immunodeficiency syndrome	NA
19	24	M	Primary immunodeficiency syndrome	NA

*M*, male; *F*, female; *CPA*, cyclophosphamide; *THP*, pirarubicin; *VDS*, vindesine; *PSL*, prednisolone; *R*, rituximab; *DXR*, doxorubicin; *VCR*, vincristine; *ETP*, etoposide; *DEX*, dexamethasone; *Bzb*, bortezomib; *NA*, not administered.

## Discussion

The present study clarified the characteristics of PML cases in Japan based on clinical data obtained through the laboratory testing for JCV DNA in CSF specimens. Mass screening of PML patients has not been feasible in Japan due to the lack of a suitable database for PML. The current strategy deals with a relatively small number of patients but has a distinct advantage in collecting precise real-time information for patients as well as specimens. The testing was constantly requested by the physicians via websites, despite the fact that there were at least 4 commercial laboratories providing similar assays during the study period according to our own survey. Thus, this internet-based approach is thought to be useful for sampling data for rare infectious diseases. In addition, as this diagnostic support system was conducted regardless of patient age, gender, underlying disease or medical history, precise information could be obtained not only from PML patients but also from CSF-JCV-negative individuals with similar conditions. These data are considered to be valuable for the examination of the overall background to PML in Japan.

A large number of PML patients had HIV infection / acquired immunodeficiency syndrome (AIDS) or hematologic disorders. Recent database analyses and other clinical studies in the USA have suggested that approximately 79–82% of PML patients are positive for HIV and 7.7–13% have hematological malignancies [24,26,31]. In contrast, the proportion of HIV-related PML cases in Japan was approximately 33%, which is much lower than that in the USA. The difference in the ratios of HIV-related PML between these two countries must be interpreted based on the epidemiological status of HIV infection. According to the latest data from the Joint United Nations Programme on HIV/AIDS, World Health Organization [32], the prevalence of HIV infection among adults in the USA (0.6%) is at least 6-fold higher than that in Japan (< 0.1%). Thus, it is reasonable to suppose that the relatively low proportion of HIV-related PML in Japan is associated the low prevalence of HIV infection. As a large proportion of HIV-infected individuals in Japan are male [32], it is also reasonable that the sex ratio of HIV-related PML showed a predominance of males.

A notable finding of the present study is that hematologic disorders are a main risk factor for PML in Japan. Five of 19 patients in this group had received allogeneic HSCT, suggesting that this type of transplantation is an important risk factor of PML. In the other 14 PML cases, 11 individuals (patients 6–16) were administrated with chemotherapeutic and / or immunosuppressive agents for the treatment of hematologic malignancies. Thus, it is likely that these therapies are associated with the high incidence of PML cases in this category. The present study also demonstrates that the majority of PML patients with hematologic disorders are males. In contrast, the percentages of male patients with hematologic malignancies were similar to or slightly higher than those of females (leukemia, 59.1%; lymphoma, 52.9%; MM, 52.1%) according to the most recent statistics from the National Database for Cancer Incidence in Japan [33]. The reason for the male predominance among PML patients with hematologic disorders remains unknown. Further studies are needed on larger populations of PML patients to clarify the mechanism and significance of this sexual dimorphism. However, these data are thought to be beneficial for patients having similar underlying diseases.

In 50 subjects with autoimmune disorders, 3 SLE patients were diagnosed as having PML. These patients had been treated with immunosuppressive agents, such as tacrolimus, mesalazine, mycophenolate mofetil, prednisolone, and / or cyclophosphamide, but not with therapeutic antibodies. No PML cases were observed among individuals with other types of autoimmune disorders. In Japan, natalizumab and efalizumab are not currently approved for use, and rituximab is not licensed for the treatment of autoimmune disorders. Therefore, increased awareness may be needed about the potential for PML in accordance with the wide spread use of therapeutic monoclonal antibodies in this country. It was also shown that the occurrence of PML is uncommon in individuals receiving solid-organ transplantation. Among the total study population, only 10 subjects underwent kidney, liver, or heart transplantation, and PML developed in one liver-transplanted patient. As this patient had suffered from common variable immunodeficiency, the association between the transplantation and PML remains unclear. This situation can be explained by the limited number of patients, who underwent organ transplantation, especially from brain dead donors [34]. However, it is predicted that the risk of PML will increase in accordance with the revision of the transplantation law in 2010, which extends the availability of transplantation therapy [35].

## Conclusions

The results of this study suggest that the internet-assisted laboratory surveillance system might be a useful strategy for elucidating the characteristics of PML on a national level. The present database provides important background information for the diagnosis and treatment of patients with risk factors for PML in Japan.

## Abbreviations

PML: Progressive multifocal leukoencephalopathy; JCV: JC virus; HIV: Human immunodeficiency virus; SLE: Systemic lupus erythematosus; CSF: Cerebrospinal fluid; NIID: National institute of infectious diseases; MRI: Magnetic resonance imaging; VP1: Viral protein 1; cART: Combination antiretroviral therapy; CD4: Cluster of differentiation 4; AA: Aplastic anemia; MM: Multiple myeloma; HSCT: Hematopoietic stem cell transplantation; AML: Acute myeloid leukemia; ALL: Acute lymphoblastic leukemia; HL: Hodgkin’s lymphoma; NHL: Non-Hodgkin’s lymphoma; AIDS: Acquired immunodeficiency syndrome.

## Competing interests

The authors declare that they have no competing interests.

## Authors' contributions

KN conceived of the study, carried out real-time PCR testing, and created the database of patients. KN and MS analyzed the clinical data and drafted the manuscript. HM and MY supervised the PML surveillance program in Japan. SK and YM participated in the clinical study of PML cases. TS performed the statistical analyses. TT supported the internet-assisted support system for JCV testing. HM, MY, SK, YM, TS, TT, CKL, and IK participated in the study design and coordination, and helped to draft the manuscript. All authors read and approved the final manuscript.

## Pre-publication history

The pre-publication history for this paper can be accessed here:

http://www.biomedcentral.com/1471-2377/12/121/prepub

## Supplementary Material

Additional file 1 **Figure S1.** Schematic diagram of the standard DNA and primer / probe sets for PCR testing. Yellow and grey lines represent the sequences of the JCV genome and pBR322 vector within the standard DNA (pJC1-4->pJCV), respectively. The numbers in the circle correspond to the nucleotide positions within the JCV genome. Three primer / probe sets detect the JCV T and VP1 genes and the boundary sequence of the JCV genome and pBR322 (green, red, and blue, respectively). Click here for file

Additional file 2 **Figure S2.** Examples of real-time PCR amplifications. Three real-time PCR assays were designed to detect the JCV T (A) and VP1 (B) sequences and the contamination of samples with standard DNA (C). The reactions were performed in the absence or presence of standard DNA (2.0 x 10^8^ to 0.8 copies per reaction). Relative fluorescence is plotted against cycle number. These PCR assays were capable of detecting at least 4 copies of JCV DNA per reaction under the same conditions. The data are representative of three independent experiments. Click here for file
