# Supplementary figures and images for: Characteristics of progressive multifocal leukoencephalopathy clarified through internet-assisted laboratory surveillance in Japan
Source: BMC Neurol. 2012 Oct 15;12:121. doi: 10.1186/1471-2377-12-121 (PMC3495800; doi:10.1186/1471-2377-12-121)

**Fig. S1**

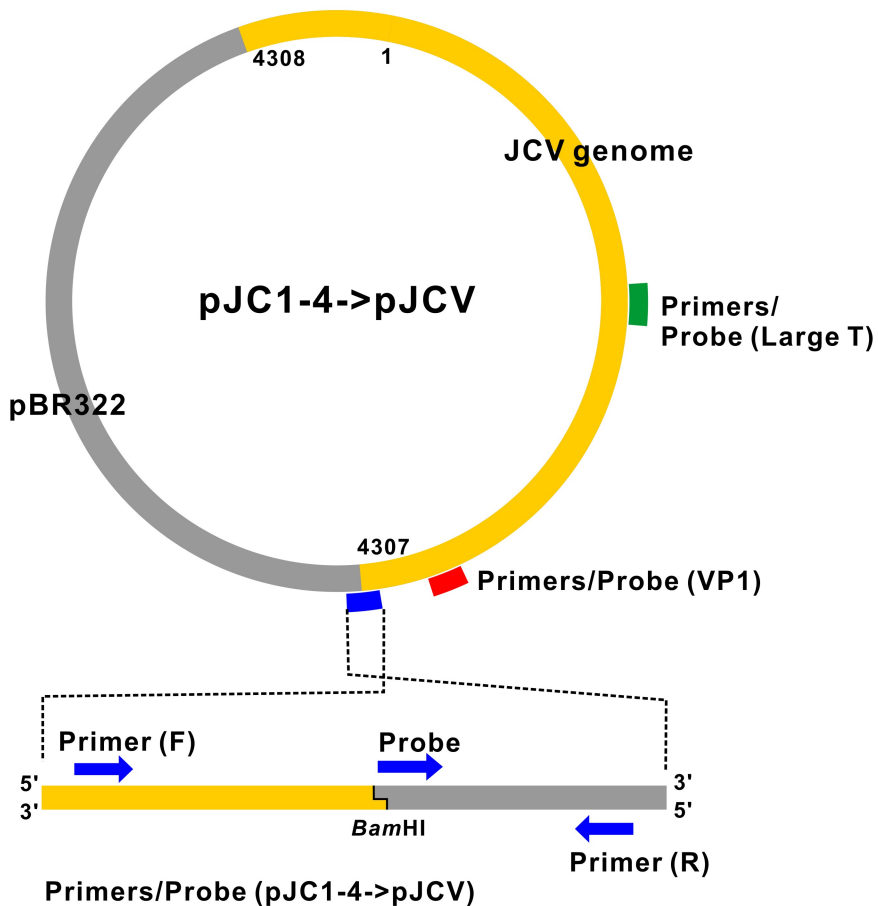

Supplement: Additional file 1 — Figure S1. Schematic diagram of the standard DNA and primer / probe sets for PCR testing. Yellow and grey lines represent the sequences of the JCV genome and pBR322 vector within the standard DNA (pJC1-4->pJCV), respectively. The numbers in the circle correspond to the nucleotide positions within the JCV genome. Three primer / probe sets detect the JCV T and VP1 genes and the boundary sequence of the JCV genome and pBR322 (green, red, and blue, respectively). [file 1471-2377-12-121-S1.pdf]

**Fig. S2**

**A**

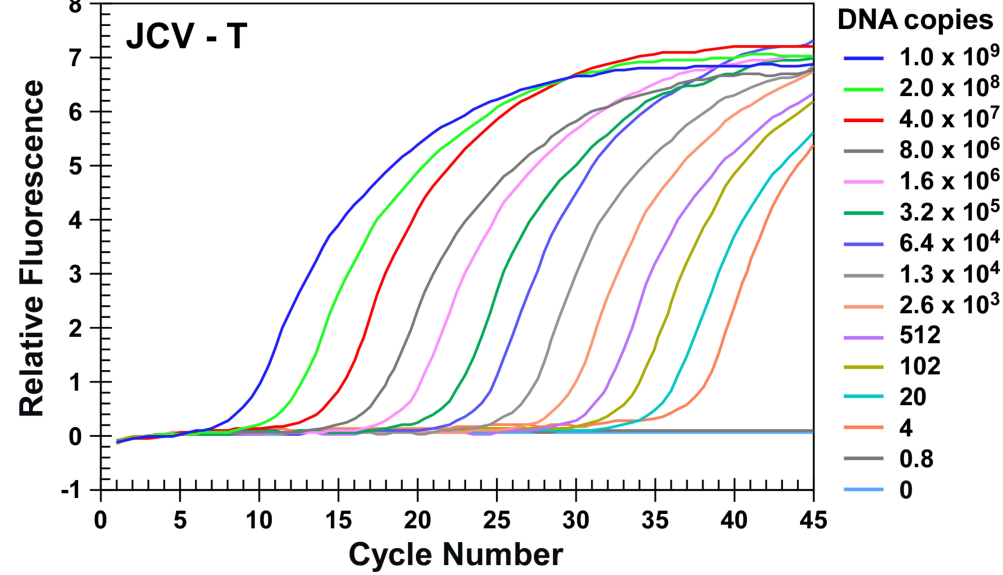

**B**

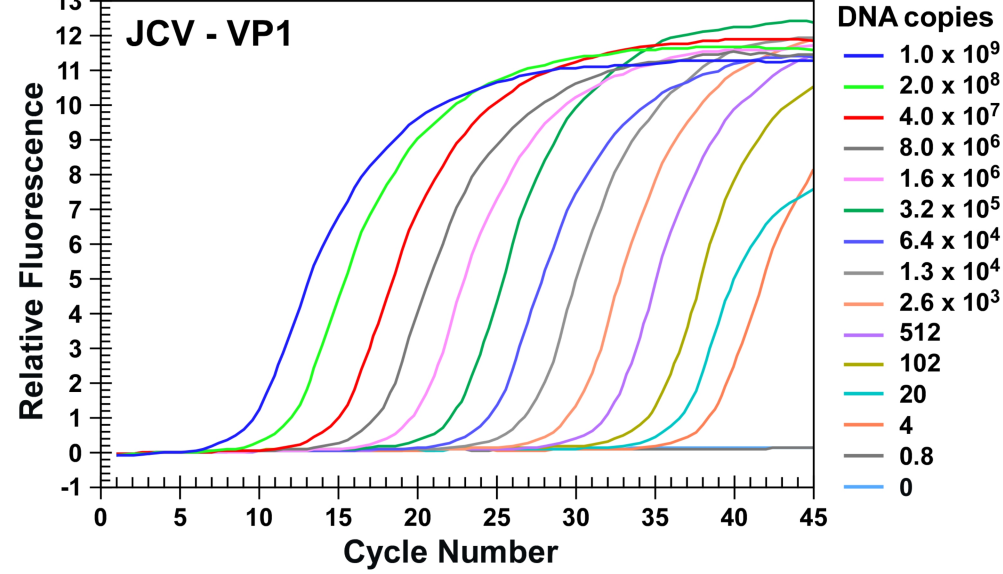

**C**

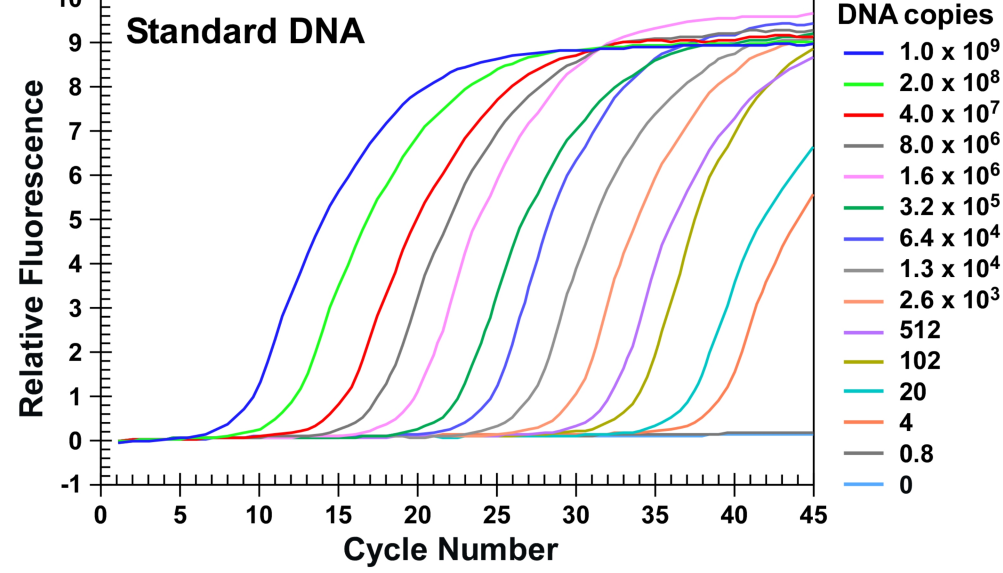

Supplement: Additional file 2 — Figure S2. Examples of real-time PCR amplifications. Three real-time PCR assays were designed to detect the JCV T (A) and VP1 (B) sequences and the contamination of samples with standard DNA (C). The reactions were performed in the absence or presence of standard DNA (2.0 x 10^8^ to 0.8 copies per reaction). Relative fluorescence is plotted against cycle number. These PCR assays were capable of detecting at least 4 copies of JCV DNA per reaction under the same conditions. The data are representative of three independent experiments. [file 1471-2377-12-121-S2.pdf]
